# Self-reported and tracked nighttime smartphone use and their association with overweight and cardiometabolic risk markers

**DOI:** 10.1038/s41598-024-55349-2

**Published:** 2024-02-28

**Authors:** Thea Otte Andersen, Christoffer Sejling, Andreas Kryger Jensen, Agnete Skovlund Dissing, Elin Rosenbek Severinsen, Henning Johannes Drews, Thorkild I. A. Sørensen, Tibor V. Varga, Naja Hulvej Rod

**Affiliations:** 1 Section of Epidemiology, Department of Public Health, University of Copenhagen, Copenhagen, Denmark; 2 Section of Biostatistics, Department of Public Health, University of Copenhagen, Copenhagen, Denmark; 3 Real World Evidence & Epidemiology, Department of Value Evidence and Patient Insights, H. Lundbeck A/S, Copenhagen, Denmark

**Keywords:** Epidemiology, Risk factors, Biomarkers

## Abstract

Nighttime smartphone use is associated with sleep problems, which in turn have a bidirectional association with overweight. We aim to investigate whether nighttime smartphone use and sleep are related to overweight and metabolic dysfunction in adult populations. We used data from three population samples (aged 16–89) from the *SmartSleep Study,* which included survey data (N = 29,838), high-resolution tracking data (N = 3446), follow-up data (N = 1768), and cardiometabolic risk markers (N = 242). Frequent self-reported nighttime smartphone use was associated with 51% higher odds (95% CI: 1.32; 1.70) of overweight compared with no use. Tracked nighttime smartphone use was also associated with overweight. Similar results were found for obesity as an outcome. No consistent associations were found between nighttime smartphone use and cardiometabolic risk markers in a small subsample of healthy young women. Poor sleep quality (vs. good sleep quality) was associated with overweight (OR = 1.19, 85% CI: 1.10; 1.28). Overall, frequent nighttime smartphone use was consistently associated with overweight and a higher BMI across diverse population samples. The bidirectional interplay between nighttime smartphone use, sleep, and overweight may create a vicious circle of metabolic dysfunction over time. Therefore, nighttime smartphone use may be a potential target point for public health interventions to reduce overweight at the population level.

## Introduction

The past decades have witnessed a steep increase in the prevalence of overweight and obesity among children, adolescents, and adults^1^. It has been estimated that 39% of adults worldwide are overweight and 13% are obese^2^. This trend is concerning, as overweight, obesity, and metabolic dysfunction are associated with an increased risk of developing diabetes, cardiovascular diseases, various types of cancer, and premature death^3,4^. Thus, identifying modifiable risk factors for overweight and obesity are essential for targeted preventive actions.

Sleep problems have been rising in parallel with the prevalence of overweight and obesity in adult populations^5,6^, and sleep and metabolism have been suggested to be linked in a bidirectional fashion^7,8^. Sleep plays a crucial role in physiological functioning, and sleep disruption may lead to metabolic dysregulation through the hyperactivation of the hypothalamic–pituitary–adrenal (HPA) axis, alterations in neuroendocrine responses, and changes in glucose metabolism^9–11^. Thus, sleep disruption may affect the control of blood glucose levels, decrease insulin sensitivity, impair β-cell function^6^, and thereby lead to metabolic dysfunction later in life. Observational studies have consistently shown that poor and short sleep is associated with higher body mass index (BMI), metabolic dysfunction, increased visceral adipose tissue, and elevated levels of cholesterol and triglycerides^8,12–18^. This is supported by evidence from experimental studies showing that sleep restriction led to changes in appetite-regulating hormones, increased caloric intake, weight changes, and alterations in glucose metabolism^19^. At the same time, obesity and obesogenic behavior may contribute to disturbed sleep^20,21^, resulting in a bidirectional relationship.

The massive and increasing around-the-clock use of smartphones constitutes one of the most pronounced behavioral changes we are experiencing today. Nighttime smartphone use is particularly prevalent in adolescents and young adults^22^. Nighttime smartphone use refers to smartphone use during sleep hours and has been associated with poor sleep quality and shorter sleep duration^22–24^. Poor sleep has been shown to mediate the effects of nighttime smartphone use on obesity^25^. Bright light exposure from the smartphone screen is associated with reduced secretion of melatonin and disruption in the circadian rhythm^26^, which may alter energy metabolism, and thus be involved in the etiological mechanisms underlying obesity^27–31^. For instance, a reduction in melatonin may impair the regulation and circadian distribution of several physiological and behavioral processes involved in energy metabolism^28^.

A smaller Danish study utilized high-resolution smartphone tracking data among young adults to measure nighttime smartphone use and found that frequent nighttime smartphone use was associated with a higher self-reported BMI^32^. Otherwise, the majority of empirical studies on this topic have been restricted to children or adolescents and used self-reported data on nighttime smartphone use^25,33–38^. These studies showed inconsistent results and may not be generalizable to adult populations. Moreover, several studies have investigated the association between overall smartphone use and overweight among adolescents or students^35,39–43^. Findings from these studies were more consistent, showing that higher overall smartphone use is associated with overweight. However, these findings may also not be generalized to adult populations. Furthermore, early adulthood is a critical period for lifelong weight trajectories, with an increased risk of excessive weight gain, and the development of obesity and metabolic dysfunction in later adulthood^44–46^.

In this project, we aim to investigate the complex relationship between nighttime smartphone use, sleep disturbances, overweight, and metabolic dysfunction. We will comprehensively assess whether self-reported and tracked nighttime smartphone use and sleep quality are related to overweight and a higher BMI in the Danish adult population. Furthermore, we will investigate whether nighttime smartphone use is associated with changes in BMI approximately 18 months later. We will also assess the association between nighttime smartphone use and cardiometabolic risk markers in a smaller sample of young adults.

## Materials and methods

### The SmartSleep Study

**Figure 1:**
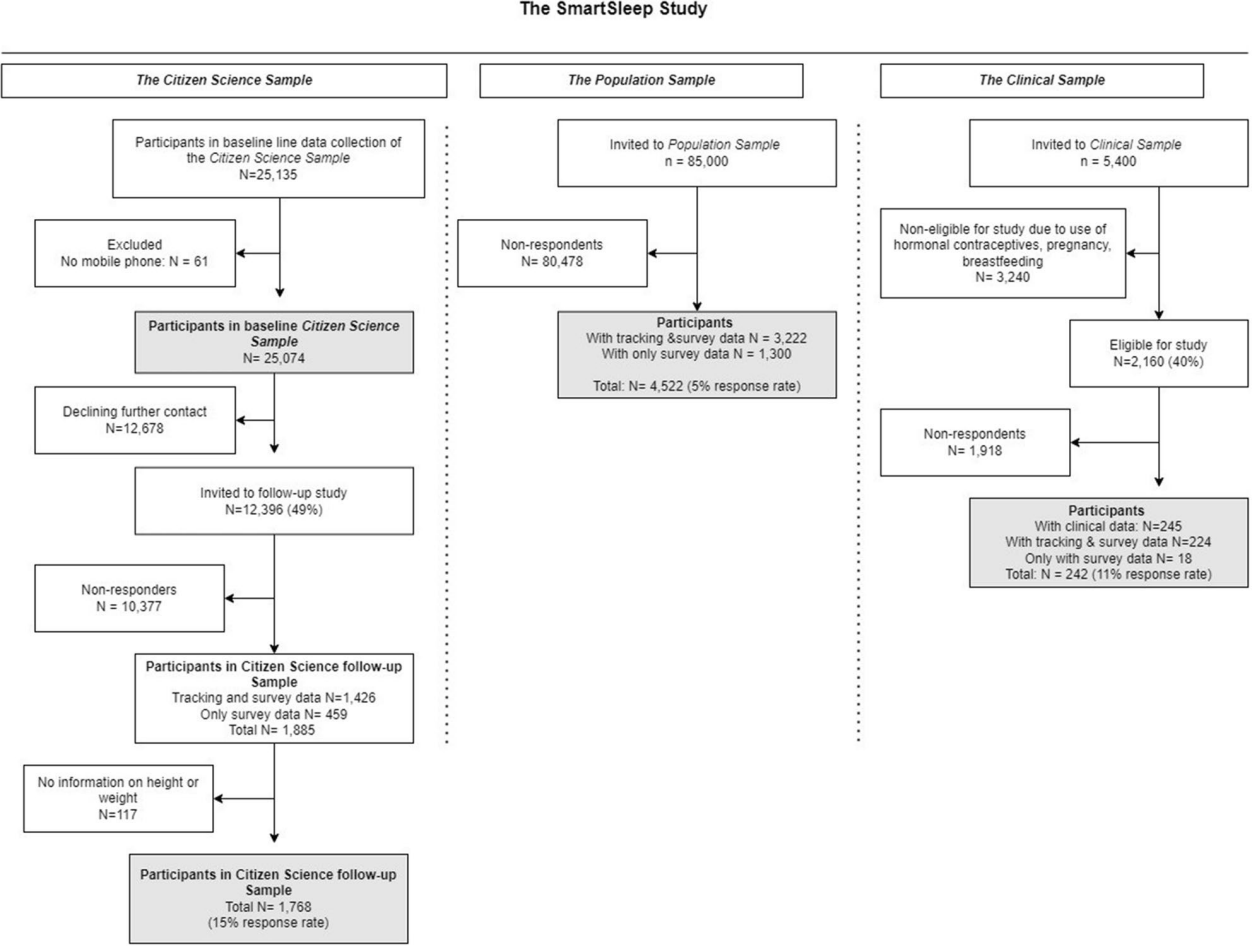
Flowchart of the three samples in the *SmartSleep Study.*

We utilized data from the *SmartSleep Study*, established in 2018, to study patterns of nighttime smartphone use in adult populations. The *SmartSleep Study* consists of three samples: the *Citizen Science Sample*, the *Population Sample*, and the *Clinical Sample,* as depicted in Fig. 1. The samples are described in detail elsewhere^47^. Briefly, the three interconnected samples employ different recruitment strategies but share overlapping measurement methods to study various aspects of nighttime smartphone use, sleep, and health.

We used data from the *Citizen Science Sample* to investigate whether self-reported nighttime smartphone use was related to BMI and its changes over 18 months. Using a citizen science approach to recruit participants, we recruited a total of 25,135 Danish adults aged 16 years and above who provided survey information at baseline. The data collection has been described elsewhere^22^. Participants with no mobile phone (N = 61) were excluded leaving 25,074 individuals eligible for the analyses. Approximately 18 months later, a total of 1,885 participants (15% response rate) participated in a follow-up study. To investigate changes in BMI, we excluded participants with missing information on height or weight at baseline or follow-up (N = 117), leaving 1768 individuals eligible for the longitudinal analysis.

We used data from the *Population Sample* to investigate associations between self-reported and latent clusters of tracked nighttime smartphone use and BMI. The data collection has been described elsewhere^47^. Participants were asked to download the *SmartSleep* app (GitHub repository: https://github.com/smartsleepku), track their nighttime smartphone use for up to 14 nights, and complete a detailed survey integrated into the app. Up to two reminders were sent to non-responders. In total, 3222 adults aged 18–50 provided both survey and tracking data, while 1300 individuals provided only survey information. A total of 4522 (5% response rate) participated in this study.

We used data from the *Clinical Sample* to explore whether nighttime smartphone use was associated with cardiometabolic risk markers, This sample was established to obtain clinical and biological measures associated with nighttime smartphone use in young female adults. A total of 245 women (11% response rate) signed informed consent and underwent a comprehensive health examination at Hvidovre Hospital, Copenhagen. The participants were also asked to download the *SmartSleep* app, track their nighttime smartphone use for up to 14 nights, and complete a survey. A total of 224 women provided both tracking and survey information, while 18 only provided survey information. Participants received DKK 500 (approx. € 67) as compensation for their participation in the study. This study was approved by the Regional Committee on Health Research Ethics (approval no: 67074).

The Danish Data Protection Agency approved all samples through the joint notification of The Faculty of Health and Medical Sciences at The University of Copenhagen (approval no. 514-0237/18-3000, 514-0288/19-3000, and 514-0344/19-3000). All methods in this study were performed following relevant guidelines and regulations.

## Data types and measurements

### Self-reported sleep quality

Sleep quality was assessed using the Danish translation of the sleep quality dimension of the validated Karolinska Sleep Questionnaire (KSQ)^48^. Sleep quality was measured based on four questions: difficulty falling asleep, disturbed and restless sleep, repeated awakenings with difficulties falling asleep again, and premature awakenings. Each item was rated on a five-point Likert scale, ranging from 1 (never) to 5 (every night or almost every night). The four items were combined into a scale ranging from 1 to 5, reflecting the average frequency of symptoms related to poor sleep quality. A higher score indicates poorer sleep quality. Sleep quality was categorized based on quartiles (Q1, Q2, Q3, Q4) in each sample.

### Self-reported nighttime smartphone use

Self-reported nighttime smartphone use was assessed by asking how often the smartphone was used after sleep onset and before sleep offset within the past three months. The response options included* “*every night or almost every night,” “a few nights a week,” “a few nights a month,” and “never”. In the questionnaire, a definition of smartphone use was provided and referred to both short and long activations of the smartphone, from simply turning on the screen to long-term use of applications.

### Clusters of nights with screen activity

The *SmartSleep app* continuously tracked all screen activations during the self-reported sleep period for up to 14 nights. We collected high-resolution smartphone tracking data on smartphone use during the sleep period over repeated nights in all three samples. Utilizing 803,000 data points in 5927 individuals with high-resolution smartphone tracking data, we used non-parametric Functional Data Analysis^49^ to identify latent clusters of nights with screen activity that characterized distinct night usage patterns*.* The development of these night clusters is described elsewhere^47^. In brief, four night clusters were identified: (1) *non-use,* defined as no smartphone activity during the self-reported sleep period, (2) *sleep onset use,* defined as smartphone activity during the sleep period mainly confined to the period right after sleep onset (the beginning of the sleep period), (3) *sleep offset use,* defined as smartphone activity during the sleep period mainly confined to the period right before sleep offset (the end of the sleep period), and (4) *continuous use,* defined as continuous smartphone activity throughout the sleep period.

### Clusters of individuals’ nighttime smartphone use

Each individual experiences a sequence of the four aforementioned night clusters that describe the different types of nights with screen activity. We utilized these sequences to cluster each individual into latent clusters using a mixture of first order Markov models^50^. To identify the number of latent clusters of individuals, we used the Expectation–maximization (EM) algorithm^51^ using the *seqHMM* R package^50^. We identified the following four latent clusters of individuals: (1) *the non-user,* characterized by most *non-use* nights. (2)* The sleep onset user,* characterized by most nights of *sleep onset use* and some nights of *non-use*. (3) *The sleep offset user,* characterized by most nights of *sleep offset use,* and (4) *the all-time user,* characterized by having a mix of night clusters with *sleep onset use, sleep offset use,* and *continuous use*. We obtained posterior probabilities of belonging to each cluster for each individual, and individuals were assigned to the clusters according to the highest posterior probability. Thus, 39**%** of the participants were assigned to the *non-user* cluster, 21% of the participants were assigned to the *sleep onset use* cluster, 27% were assigned to the *sleep offset user* cluster, and 13% were assigned to the *all-time user* cluster. For more information about the four latent clusters of individuals’ nighttime smartphone use, please see Supplementary Text S1.

### Body Mass Index (BMI)

In the *Population Sample* and *Citizen Science Sample*, self-reported weight was measured to the nearest kilogram, and self-reported height was measured to the nearest centimeter. BMI was calculated as weight in kilograms divided by height in meters squared. In the *Clinical Sample*, a professional measured weight and height at the health examination and calculated the same way as above. More information on how BMI was measured is given in Supplementary Table S1. Overweight and obesity were defined as BMI values ≥ 25 kg/m^2^ and ≥ 30 kg/m^2^, respectively, according to the World Health Organization guidelines^52^.

### Cardiometabolic risk markers

At the health examination in the *Clinical Sample,* waist and hip circumference as well as blood pressure were measured for all women. Plasma from non-fasting venous blood samples was collected using K2-EDTA (for HbA1c) and Li-heparin (for all other biomarkers) tubes. The samples were analyzed on the same day at the Department of Clinical Biochemistry at Copenhagen University Hospital Hvidovre (Cobas 8000, TOSOH HLC—723 G8). The biomarkers measured from fresh plasma included *triglycerides*, *high-density lipoprotein cholesterol* (HDL-C), *low-density lipoprotein cholesterol* (LDL-C), *very-low-density lipoprotein cholesterol* (VLDL-C), *total cholesterol (*TC*),* and *glycated hemoglobin (*HbA1c). See an overview of the cardiometabolic risk markers in Supplementary Table S1.

### Covariates

Information on *age*, *gender* (male, female, other), *educational level* (primary school, upper secondary school, technical/vocational education, short-cycle higher education, medium-cycle higher education, long-cycle higher education, other education), and *occupational status* (student, employed, unemployed, long-term sick leave, outside labor market (retirement or early retirement), other occupation) was collected from the survey. In the *Population Sample*, we used information on biological sex (male, female) and age from the Central Person Register (CPR) in Denmark. The *Citizen Science Sample* extracted *follow-up time* based on the time difference between baseline and follow-up response times. *Physical activity* was assessed by asking the participants to characterize their level of physical activity within the past year. The response options included “high-intensity training several times a week,” “exercise or heavy gardening for at least four hours a week,” “light exercise for at least four hours a week,” or “doing mainly sedentary activities.”

### Statistical analysis

All analyses were undertaken using R 4.1.1. Weighting on age, sex, and geographical region in Denmark was performed using raking^53^ for the *Baseline* and *Follow-up Citizen Science Sample* and the *Population Sample* to achieve more representative population-based samples (Supplementary Table S2). No weighting was performed for the *Clinical Sample* as this sample was not established to be representative.

The missing survey data in each sample were imputed using multivariate imputation by chained equations (*mice* package in R, version 3.14.0)^54^. Random forest was employed for imputing all survey variables (N = 257), with 75 iterations, and 25 imputed copies were generated for each sample. The imputation of data for the *Citizen Science Sample* and the *Population Sample* incorporated the sampling weights to account for the complex survey design^55^. Convergence was visually inspected for randomly selected imputed datasets. All downstream analyses were undertaken in each of the 25 imputed datasets, and results were subsequently pooled using Rubin’s rule^56^. Confidence intervals for model parameters and *P* values for the statistical tests were obtained using Wald-type calculations.

To describe the samples, means and standard deviations (SD) were reported for continuous variables, and frequencies and percentages were reported for categorical variables.

We investigated the associations between sleep quality, self-reported and latent clusters of nighttime smartphone use, and BMI and cardiometabolic risk markers. Logistic regression models were fitted for overweight and obesity as outcomes, and odds ratios (OR) and 95% confidence intervals (95% CI) were reported (*stats* package in R, version 4.2.1). Age, gender/sex, educational level, and occupational status were identified as potential confounders and were adjusted for in all models. In the sleep quality model, we further adjusted for nighttime smartphone use. Results from the *Citizen Science Sample* and the *Population Sample* for the association between sleep quality, self-reported nighttime smartphone use, and overweight/obesity were pooled using inverse variance weighted fixed-effects meta-analysis (*meta* package in R, version 6.0-0), as we expected that the samples were estimating the same effect size as the samples were rather homogenous in terms of study populations, study designs, and measurements.

When considering continuous variables as an outcome, we used linear regression models and the Generalized Additive Models for Location Scale and Shape (GAMLSS), a semi-parametric model which allows all distribution parameters to be modeled flexibly as functions of explanatory variables (gamlss package in R, version 5.4-3)^57,58^. The Box-Cox-Cole and Green (BCCG) model from the GAMLSS regression framework was used^58^. See Supplementary Text S2 for further details on the analyses. All models were adjusted for age, gender/sex, educational level, and occupational status.

For participants in the *Citizen Science Sample* with follow-up information (N = 1768), we explored whether the self-reported nighttime smartphone use was associated with changes in BMI over 18 months using GAMLSS models. Changes in BMI (in kg/m^2^) were calculated as the mean difference in BMI between baseline and follow-up. We accounted for individual differences in follow-up time by adjusting for follow-up time and including an interaction between nighttime smartphone use and follow-up time. Furthermore, all models were adjusted for age, gender/sex, educational level, and occupational status.

### Sensitivity analysis

We assume that physical activity was a variable on the causal pathway from nighttime smartphone use to BMI and metabolic dysfunction. However, as the measures were reported at the same point in time, we could not determine whether physical activity may be a confounding variable. Thus, in a sensitivity analysis, we further adjusted for physical activity to assess whether physical activity confounded the association between nighttime smartphone use and overweight.

## Results

### Population characteristics

In the *Baseline Citizen Science Sample*, those with more frequent self-reported nighttime smartphone use were younger, more likely to be female and students than individuals with no nighttime smartphone use (Table 1). No differences were seen in follow-up time across self-reported nighttime smartphone use. In the *Population Sample,* similar characteristics were found as more women and students had more frequent self-reported nighttime smartphone use (Supplementary Table S3). In the *Clinical Sample,* the majority of the women were students, and approximately one in ten women (12%) used their smartphones every night or almost every night (Supplementary Table S4).

**Table 1:** Characteristics of the study population stratified on self-reported nighttime smartphone use among 25,074 in the *Baseline Citizen Science Sample.*

	Total N = 25,074	Frequency of self-reported nighttime smartphone use			
		Never N = 10,475 (42%)	A few nights a month N = 10,174 (40%)	A few nights a week N = 3206 (13%)	Every night or almost every night N = 1219 (5%)
Age, mean (SD)	42.9 (15.3)	48.7 (14.6)	39.8 (14.2)	35.9 (14.2)	36.7 (15.2)
Gender, N (%)					
Female	15,464 (62)	5848 (56)	6628 (65)	2150 (67)	838 (69)
Male	9564 (38)	4611 (44)	3529 (35)	1047 (33)	377 (31)
Other	46 (0)	16 (0.2)	17 (0.2)	9 (0.3)	4 (0.3)
Educational level, N (%)					
Primary school	1796 (7)	600 (6)	681 (7)	346 (11)	169 (14)
Upper secondary school	2777 (11)	698 (7)	1283 (13)	191 (10)	191 (16)
Technical/Vocational education	3494 (14)	1749 (17)	1270 (13)	323 (19)	152 (13)
Short-cycle higher education	1964 (8)	941 (9)	723 (7)	224 (7)	76 (6)
Medium-cycle higher education	7937 (32)	3480 (33)	3231 (32)	906 (28)	320 (26)
Long-cycle higher education	6566 (26)	2754 (26)	2778 (27)	753 (24)	281 (23)
Other	540 (2)	253 (2)	208 (2)	49 (2)	30 (3)
Occupational level, N (%)					
Student	4148 (17)	887 (5)	1975 (19)	948 (30)	338 (28)
Employed	16,275 (65)	7064 (62)	6794 (67)	1790 (56)	627(51)
Unemployed	671 (3)	241 (3)	267 (3)	111 (4)	52 (4)
Long-term sick leave	352 (1)	115 (1)	138 (1)	71 (2)	28 (2)
Outside labor market	2764 (11)	1,835 (25)	664 (7)	167 (5)	98 (8)
Other	864 (3)	333 (3)	336 (3)	119 (4)	76 (6)
Follow-up time, year (SD)	1.49 (1.46–1.53)	1.48 (1.46–1.53)	1.49 (1.47–1.53)	1.49 (1.46–1.53)	1.49 (1.47–1.53)

### Sleep and overweight

**Figure 2:**
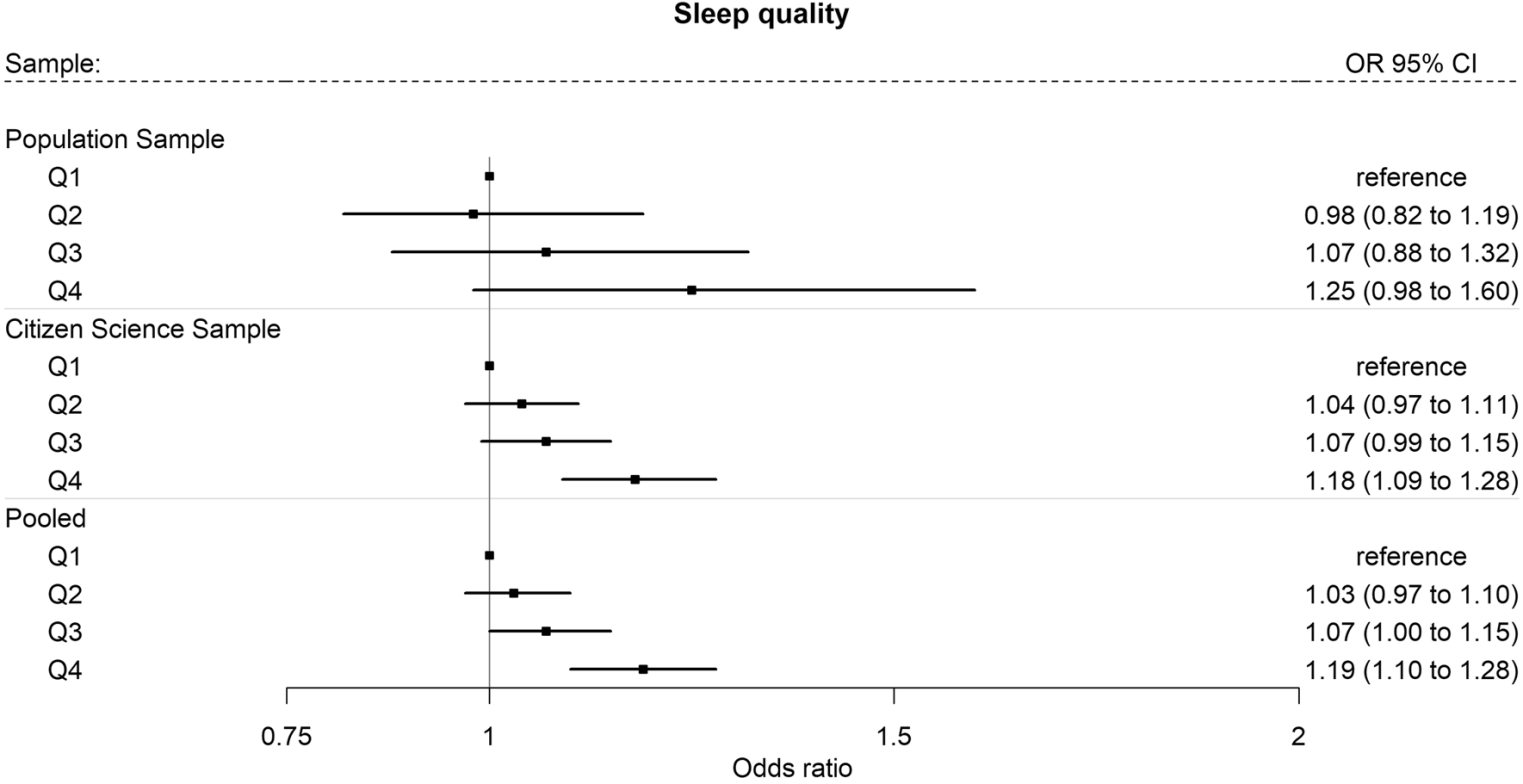
Associations between sleep quality and overweight^1^ in the Population Sample (N = 4522) and the Citizen Science Sample (N = 25,074) and pooled^2^ in a fixed-effect meta-analysis. ^1^Outcome variable: overweight defined as BMI ≥ 25. ^2^Estimates pooled across the *Citizen Science Sample* and the *Population Sample.* Logistic regression models adjusted for age, gender/sex, educational level, occupational status, and nighttime smartphone use were applied. Models were weighted by sample weights for *Population Sample* and *Citizen Science Sample.* Fixed effect inverse variance weighted meta-analysis*:*Heterogeneity*: Q2:* I^2^ = 0%, Cochrane’s Q p = 0.56, Q3: I^2^ = 0%, Cochrane’s Q p = 0.61, Q4: I^2^ = 0%, Cochrane’s Q p = 0.66.

We found that individuals with the poorest sleep quality (Q4) were associated with 19% higher odds (OR = 1.19, 95%CI: 1.10; 1.28) of overweight compared with individuals with good sleep quality (Q1) in the pooled analysis (Fig. 2). Similar results were found in the association between sleep quality and obesity (Supplementary Table S5).

### Nighttime smartphone use and overweight

**Figure 3:**
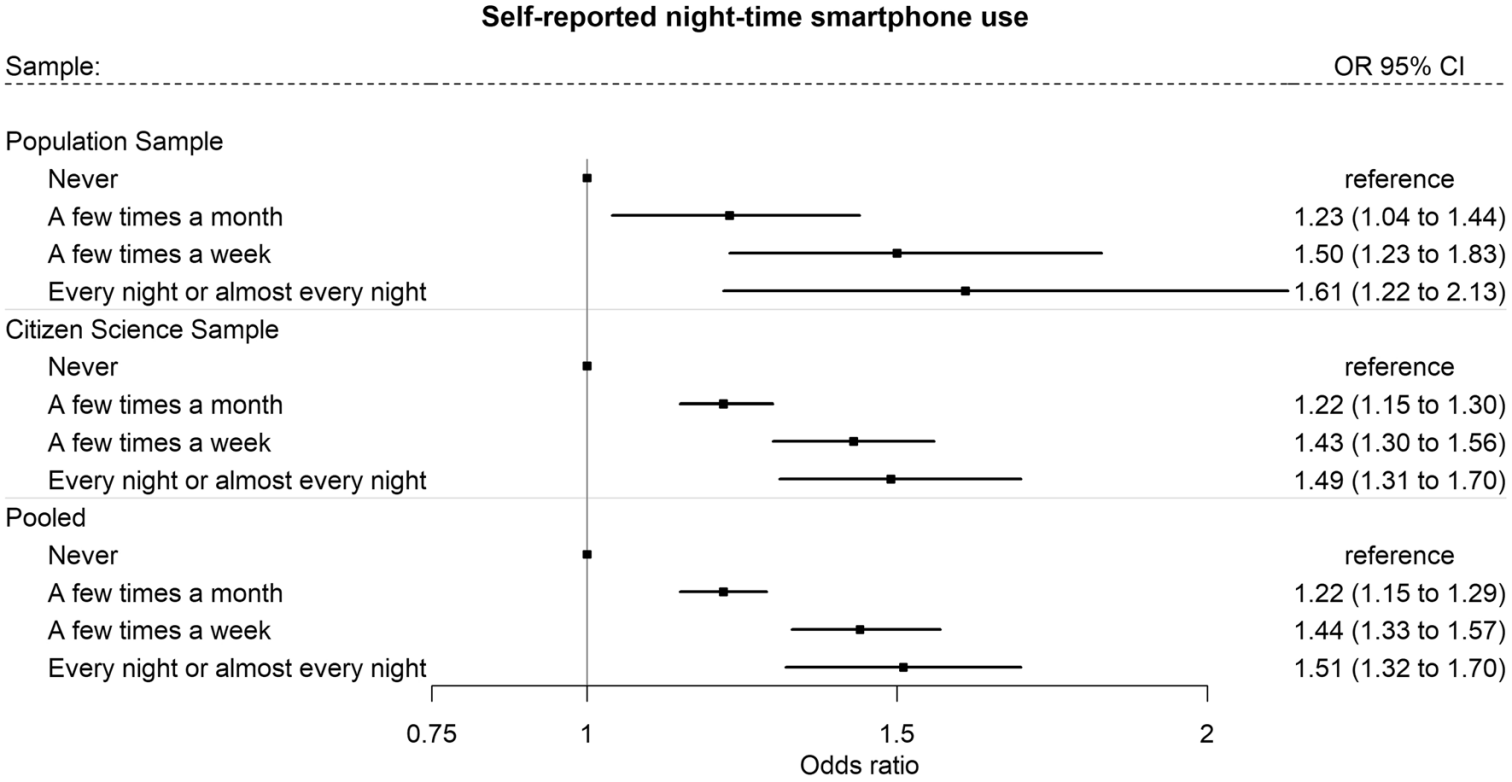
Associations between self-reported nighttime smartphone use and overweight^1^ in the *Population Sample* (N = 4522), the *Citizen Science Sample* (N = 25,074), and pooled^2^ in a fixed effect meta-analysis. ^1^Outcome variable: overweight defined as BMI ≥ 25. ^2^Estimates pooled across the *Citizen Science Sample* and the *Population Sample.* Logistic regression models adjusted for age, gender/sex, educational level, and occupational status were applied. Models were weighted by sample weights for *Population Sample* and *Citizen Science Sample.* Fixed effect inverse variance weighted meta-analysis*:* Heterogeneity*: A few times a month:* I^2^ = 0%, Cochrane’s Q p = 0.93, *A few times a week*: I^2^ = 0%, Cochrane’s Q p = 0.67, *Every night or almost every night*: I^2^ = 0%, Cochrane’s Q p = 0.62.

We found that frequent self-reported nighttime smartphone use (every night or almost every night) was associated with 49% higher odds (OR: 1.49, 95% CI: 1.31; 1.70) of overweight in the Citizen Science Sample and 61% higher odds (OR: 1.61, 95% CI: 1.22; 2.13) of overweight in the Population Sample compared to no nighttime smartphone use (Fig. 3). In the pooled analysis, we found that frequent nighttime smartphone use was associated with 51% higher odds (OR: 1.51, 95% CI: 1.32; 1.70) of overweight compared to no nighttime smartphone use.

**Figure 4:**
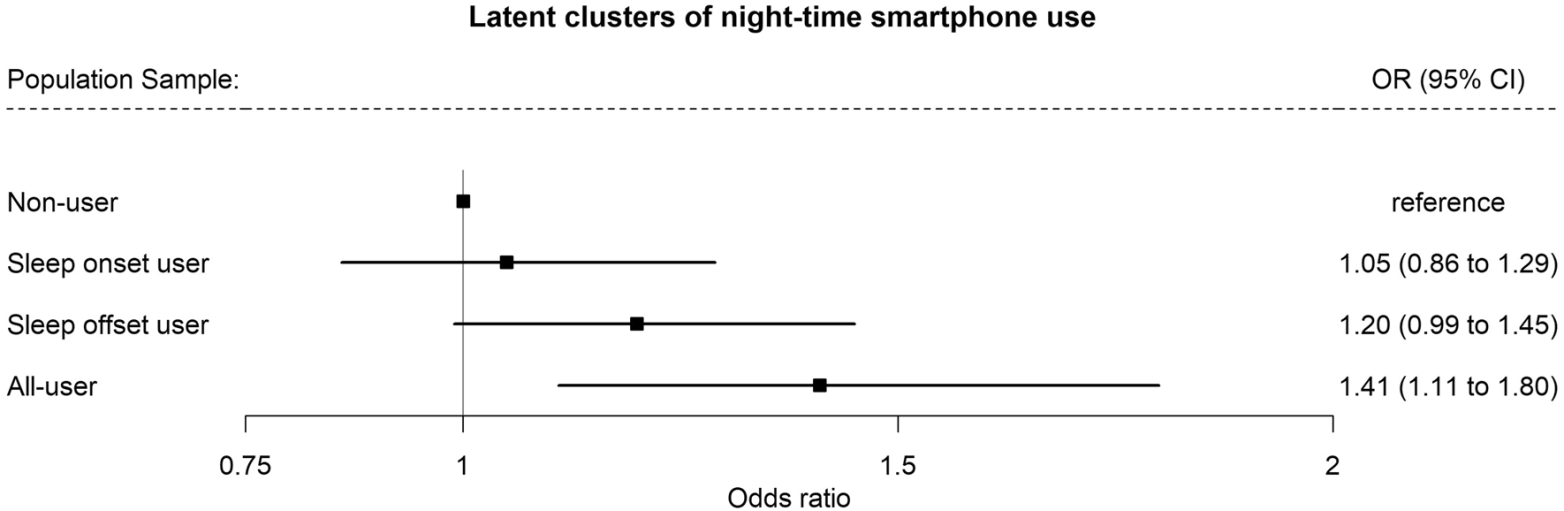
Associations between latent clusters of nighttime smartphone use and overweight in the *Population Sample* (N = 3222). ^1^Outcome variable: Overweight defined as BMI ≥ 25. Logistic regression models were adjusted for age, gender/sex, educational level, and occupational status. Weighted by sample weights for *Population Sample.*

Similar associations were found for the latent clusters of tracked nighttime smartphone use in the *Population Sample*, with the sleep offset user (OR: 1.20, 95% CI: 0.99; 1.45) and all-time user (OR: 1.41, 95% CI: 1.11; 1.80) showing higher odds of overweight compared to the non-user cluster (Fig. 4).

In the *Clinical Sample,* which included a small sample of healthy young women (N = 242), only a few participants were overweight (BMI ≥ 25 kg/m^2^), and thus, we did not explore the association between nighttime smartphone use and overweight and obesity in this sample.

When exploring the association between self-reported and tracked nighttime smartphone use and obesity (BMI ≥ 30 kg/m^2^), we found similar patterns with higher odds of obesity with more frequent nighttime smartphone use (Supplementary Table S6).

In a sensitivity analysis, we further adjusted for physical activity in the association between self-reported and tracked nighttime smartphone use and overweight in the *Population Sample*. (Supplementary Table S7). The estimates did not change markedly. Thus, we expect that physical activity did not influence our results.

### Nighttime smartphone use and BMI

Frequent self-reported nighttime smartphone use was associated with higher BMI across all three samples (Table 2). Using the smartphone every night or almost every night was associated with 0.65 (β = 0.65, 95% CI: 0.40; 0.89) point higher BMI in the *Citizen Science Sample*, 0.86 (β = 0.86, 95% CI: 0.32; 1.41) point higher BMI in the *Population Sample,* and 2.37 (β = 2.37, 95% CI: 0.64; 4.62) points higher BMI in the *Clinical Sample* compared to no nighttime smartphone use. For the latent clusters of tracked nighttime smartphone use in the *Population Sample*, the sleep onset user (β = 0.15, 95% CI: − 0.24; 0.54), the sleep offset user (β = 0.44, 95% CI: 0.07; 0.81), and the all-time user (β = 0.31, 95% CI: − 0.17; 0.78) were associated with higher BMI compared to the non-user cluster. In the *Clinical Sample*, the association between the latent clusters of tracked nighttime smartphone use and BMI was less consistent.

**Table 2:** Associations between self-reported and tracked nighttime smartphone use and Body Mass Index (BMI) in the three samples.

	Citizen science sample N = 25,074	Population sample N = 4522^a^	Clinical sample N = 242^a^
	BMI (kg/m^2^)		
	β (95%CI)	β (95%CI)	β (95%CI)
Self-reported nighttime smartphone use			
Never	Ref.	Ref.	Ref.
A few nights a month	0.41 (0.29; 0.52)	0.33 (0.03; 0.64)	0.15 (− 1.39; 1.69)
A few nights a week	0.66 (0.50; 0.83)	0.61 (0.23; 0.99)	0.34 (− 1.30; 1.98)
Every night or almost every night	0.65 (0.40; 0.89)	0.86 (0.32; 1.41)	2.37 (0.44; 4.62)
Latent clusters of tracked nighttime smartphone use			
Non-users	*–*	Ref.	Ref.
Sleep onset users	*–*	0.15 (− 0.24; 0.54)	− 0.51 (− 1.81; 0.79)
Sleep offset users	*–*	0.44 (0.07; 0.81)	0.07 (− 1.26; 1.39)
All-time users	*–*	0.31 (− 0.17; 0.78)	0.02 (− 1.45; 1.49)

All models in the *Citizen Science Sample* and *Population Sample* were adjusted for gender/sex, educational level, and occupational status. All models in the *Clinical Sample* were adjusted for age, educational level (categorized as primary school or other; upper secondary school; technical/vocational education/short-cycle higher education; medium-cycle higher education; long-cycle higher education), and occupational status (categorized as student; employed; other).

Models were weighted by sample weights for *Population Sample* and* Citizen Science Sample.*

Generalized Additive Models for Location Scale and Shape (GAMLSS) models were applied for the analyses.

β, mean difference; 95% CI, 95% confidence interval.

^a^Latent clusters of nighttime smartphone use: Population Sample: N = 3222, Clinical Sample: N = 224. No smartphone tracking data were collected in the *Baseline Citizen Science Sample.*

### Nighttime smartphone use and changes in BMI

Table 3 shows the longitudinal changes in BMI over 18 months in the *Citizen Science Follow-up Sample*. On average, the mean differences in BMI for women and men were 0.32 kg/m^2^ and 0.13 kg/m^2^, respectively. We found that frequent self-reported nighttime smartphone use (every night or almost every night) was associated with a 0.24-point higher BMI change (β = 0.24, 95% CI: − 0.02; 0.50) compared to the BMI change in the reference group. However, the 95% confidence intervals overlapped the null.

**Table 3:** Changes in Body Mass Index (BMI) over 18 months among 1768 participants in the *Follow-up Citizen Science Sample.*

	Changes in BMI^a^ β kg/m^2^ (95% CI)
Self-reported nighttime smartphone use at baseline	
Never^b^	Ref.
A few nights a month	0.03 (− 0.10; 0.15)
A few nights a week	0.18 (− 0.01; 0.37)
Every night or almost every night	0.24 (− 0.02; 0.50)

Models were weighted by sample weights for F*ollow-up Citizen Science Sample.*

Generalized Additive Models for Location Scale and Shape (GAMLSS) models adjusted for gender, age, educational level, occupational status, and follow-up time were applied. An interaction between nighttime smartphone use and follow-up time was included in the model.

β, mean difference; 95% CI, 95% confidence interval.

^a^Difference between BMI at baseline and BMI at follow-up.

^b^For reference group: Average BMI change: 0.14 kg/m^2^.

### Self-reported and tracked nighttime smartphone use and cardiometabolic risk markers

Table 4 shows the cross-sectional associations of nighttime smartphone use with cardiometabolic risk markers in a small clinical sample of healthy young women. For the reference group with no self-reported nighttime smartphone use, the average level for each cardiometabolic risk marker was within the normal range. We found that both systolic blood pressure (A few times a week: β = 1.68, 95% CI; − 2.93; 6.29 and Every night or almost every night: β = 1.62, 95% CI: − 3.80; 7.05) and diastolic blood pressure (A few nights a week: β = 2.36, 95%CI: − 1.16; 5.88 and every night or almost every night: β = 1.25, 95%CI: − 2.90; 5.41) were higher for those with frequent nighttime smartphone use compared to those with no nighttime smartphone use, although the confidence intervals were overlapping the null. Systolic and diastolic blood pressure was also higher in latent clusters of tracked nighttime smartphone use compared to the non-user cluster, but the confidence intervals overlapped the null.

**Table 4:** Associations between self-reported nighttime smartphone use (N = 242) and clusters of tracked nighttime smartphone use (N = 224) and cardiometabolic risk markers among young women in the *Clinical Sample.*

	Self-reported nighttime smartphone use			
	Never (Ref.) N = 27 (11%)	A few nights a month N = 120 (50%)	A few nights a week N = 66 (27%)	Every night or almost every night N = 29 (12%)
	Mean (SD)	β (95% CI)	β (95% CI)	β (95% CI)
Waist-hip ratio	0.8 (0)	0.01 (− 0.01; 0.03)	0.01 (− 0.02; 0.03)	0.01 (− 0.01; 0.04)
Blood pressure (BP), mmHg				
Systolic BP	115.9 (9.6)	0.16 (− 4.16; 4.48)	1.68 (− 2.93; 6.29)	1.62 (− 3.80; 7.05)
Diastolic BP	71.3 (7.2)	1.18 (− 2.12; 4.48)	2.36 (− 1.16; 5.88)	1.25 (− 2.90; 5.41)
Lipid biomarkers, mmol/L				
Total cholesterol	4.2 (0.6)	0.06 (− 0.22; 0.35)	0.02 (− 0.29; 0.32)	− 0.03 (− 0.39; 0.33)
HDL-C	1.7 (0.4)	0.05 (− 0.10; 0.20)	− 0.01 (− 0.17; 0.16)	− 0.05 (− 0.24; 0.14)
LDL-C	2.0 (0.5)	− 0.03 (− 0.27; 0.22)	− 0.00 (− 0.27; 0.26)	− 0.03 (− 0.33; 0.28)
VLDL-C	0.4 (0.2)	0.02 (− 0.08; 0.12)	0.01 (− 0.09; 0.12)	0.02 (− 0.10; 0.15)
Triglycerides	0.9 (0.4)	0.04 (− 0.18; 0.27)	0.02(− 0.22; 0.26)	0.03 (− 0.25; 0.32)
HbA1c	31.6 (3.2)	0.87 (− 0.62; 2.37)	0.58 (− 1.04; 2.21)	0.82 (− 1.12; 2.76)

	Latent clusters of tracked nighttime smartphone use			
	Non-user (ref.) N = 74 (33%)	Sleep onset user N = 57 (25%)	Sleep offset user N = 55 (25%)	All-time user N = 38 (17%)
	Mean (SD)	β (95% CI)	β 95% CI)	β (95% CI)
Waist-hip ratio	0.8 (0.1)	− 0.01 (− 0.03; 0.01)	0.01 (− 0.01; 0.03)	− 0.01 (− 0.03; 0.01)
Blood pressure (BP), mmHg				
Systolic BP	115.7 (10.1)	2.08 (− 1.58; 5.75)	1.68 (− 2.04; 5.41)	2.22 (− 1.93; 6.36)
Diastolic BP	72.3 (7.1)	0.74 (− 2.02; 3.51)	1.22 (− 1.59; 4.03)	2.22 (− 0.91; 5.35)
Lipid biomarkers, mmol/L				
Total cholesterol	4.4 (0.6)	− 0.41 (− 0.64; − 0.17)	− 0.24 (− 0.49; − 0.00)	− 0.23 (− 0.50; 0.04)
HDL-C	1.8 (0.4)	− 0.10 (− 0.23; 0.03)	− 0.03 (− 0.16; 0.09)	− 0.11 (− 0.25; 0.04)
LDL-C	2.2 (0.5)	− 0.24 (− 0.45; − 0.04)	− 0.20 (− 0.41; 0.01)	− 0.10 (− 0.33; 0.14)
VLDL-C	0.4 (0.2)	− 0.04 (− 0.12; 0.05)	0.01 (− 0.07; 0.10)	− 0.01 (− 0.10; 0.09)
Triglycerides	1.0 (0.5)	− 0.10 (− 0.30; 0.09)	0.00 (− 0.19; 0.19)	− 0.04 (− 0.26; 0.17)
HbA1c	31.7 (2.8)	0.86 (− 0.38; 2.09)	− 0.21 (− 1.45; 1.04)	1.48 (− 0.09; 2.87)

Linear regression models adjusted for age, educational level (categorized as primary school or other; upper secondary school; technical/vocational education/short-cycle higher education; medium-cycle higher education; long-cycle higher education), and occupational status (categorized as student; employed; other) were applied.

HDL-C, high-density lipoprotein cholesterol; LDL-C, low-density lipoprotein cholesterol; VLDL-C, very low-density lipoprotein cholesterol; HbA1C, glycated hemoglobin; BP, blood pressure; ref, reference group; β, mean difference; 95% CI, 95% confidence intervals.

We found no clear associations between nighttime smartphone use and cardiometabolic risk markers of waist-hip ratio, total cholesterol, LDL-C, VLDL-C, HDL-C, triglycerides, or HbA1c, except for slightly lower levels of total cholesterol and LDL-C in the sleep onset cluster compared to the non-user cluster.

## Discussion

We aimed to investigate the complex relationship between nighttime smartphone use, sleep, and overweight and cardiometabolic risk markers, by combining different measures of nighttime smartphone use and cardiometabolic measures in three population samples. We found that poor sleep quality was associated with overweight. Moreover, self-reported frequent nighttime smartphone use was associated with overweight, obesity, and a higher average BMI across all samples. Furthermore, we identified four distinct latent clusters of nighttime smartphone use using high-resolution smartphone tracking data. We found that clusters characterized by nighttime smartphone use were associated with overweight, obesity, and a higher BMI in the *Population Sample.* However, these associations between latent clusters of nighttime smartphone use and BMI were not replicated in the smaller *Clinical Sample,* which only included healthy young women.

Only a few previous studies have addressed the association between nighttime smartphone use and overweight^32–34,38^, but these studies only included adolescents or students and were cross-sectional in design. Nevertheless, in line with findings from these studies and our hypothesis, we found a consistent association between nighttime smartphone use and overweight and obesity across population samples from the general adult population in Denmark.

Nighttime smartphone use may lead to weight gain and the eventual development of obesity via various mechanisms. First, nighttime smartphone use may impact the secretion of melatonin, a hormone involved in regulating the circadian rhythm, energy metabolism, gut microbiota, and inflammation^29–31^. Sleep problems may also mediate the relationship between nighttime smartphone use and overweight. We showed that poor sleep quality was associated with overweight, which aligns with numerous studies showing that short sleep duration and poor sleep quality are associated with behavioral, metabolic, and endocrine changes that lead to weight gain and subsequently obesity^9,16,18,19,21,59^. At the same time, nighttime smartphone use has been linked to poor and disturbed sleep^22,24^. Other mechanisms explaining the link between nighttime smartphone use and overweight may include reduced physical activity and increased energy intake due to daytime tiredness and fatigue^21,37^. These findings indicate that nighttime smartphone use may negatively impact metabolism and BMI. Thus, nighttime smartphone use may be a potential target point for preventive interventions to reduce overweight and obesity at the population level. Future intervention studies may also benefit from investigating whether specific smartphone apps (i.e., social media, streaming, gaming, or news), as well as different exposures of light intensity used during sleep hours may influence the relationship between nighttime smartphone use and overweight.

Contrary to our hypothesis, we did not find significant changes in BMI over 18 months associated with self-reported nighttime smartphone use. Similarly, a recent study among adolescents did not find associations between nighttime smartphone use and changes in weight during a two-year follow-up period^36^. These findings may question the causality of the observed cross-sectional associations between nighttime smartphone use and overweight/obesity observed in this and other studies. Nevertheless, it is important to mention that nighttime smartphone use was measured at a random time in people’s lives (maybe after e.g., ten years of nighttime smartphone use), and the associated metabolic dysfunction may therefore already have been established. Also, the 18-month period may be relatively short to investigate clinically significant weight changes. We suggest that future studies investigate the temporal association between nighttime smartphone use and changes in BMI over a longer period (and preferable from initiation of use) to elucidate the longitudinal effects of nighttime smartphone use on metabolic dysfunction.

Another possible explanation for our findings relates to reverse causality. Evidence suggests that individuals with obesity report more sleep problems than those without obesity^8,21^, and individuals experiencing sleep problems may use their smartphones during the sleep period to counteract their sleep problems or combat insomnia. Mechanisms linking obesity to sleep problems include several obesity-related factors that may interfere with normal sleep quality and sleep duration^14,20,60^. Indeed, increased visceral adipose tissue may play a role in the pathogenesis of poor sleep due to elevated levels of pro-inflammatory cytokines that may disrupt the circadian rhythm^21^. High consumption of carbohydrates, particularly in the evening, may also negatively impact sleep^21^. Thus, the bidirectional interplay between nighttime smartphone use, sleep, and overweight may create a vicious circle of metabolic dysfunction over time.

We generally found no strong associations between nighttime smartphone use and cardiometabolic risk markers. We only investigated these associations in a small study sample of healthy young women, so the general lack of associations is unsurprising. We suggest that future studies include clinical cardiometabolic measures in a larger and more representative adult study population concerning gender, age, and health.

## Strengths and limitations

The present study included data from three diverse samples, which allowed us to robustly validate and compare findings across samples in a triangulation framework^61^. Also, we explored nighttime smartphone use using self-reported and tracked measures, which have previously been found to be only moderately correlated, indicating potential underreporting in self-reported smartphone use compared to tracked smartphone use^62^. Additionally, using latent clusters of nighttime smartphone use based on high-resolution smartphone tracking data, we identified distinctive characteristics representing different dynamical patterns of nighttime smartphone use. Furthermore, we assessed metabolic dysfunction using various cardiometabolic risk markers including BMI, anthropometrics, and clinical biomarkers. Other strengths of the present study include the longitudinal study design to assess changes in BMI, multiple imputation of survey data to reduce bias introduced by missingness, and raked-weighting methodology to increase the representativeness of the study samples.

The present study also has several limitations. First, we could not make any causal conclusions based on our analytic design. Although we found consistent associations between nighttime smartphone use and overweight/obesity, we could not ascertain whether metabolic dysfunction is caused by nighttime smartphone use, vice versa, or by a common underlying cause. In the longitudinal analyses evaluating changes in BMI within 18 months, the two time points may be suboptimal for longitudinal assessments as it is cumbersome to differentiate between true change and measurement error.

In the present study, misclassification may be a potential source of bias, as we use survey data to assess self-reported nighttime smartphone use and BMI. Thus, there may be a risk of recall or social desirability bias, where participants may either under or over-report their nighttime smartphone use, height, and weight. Nevertheless, we applied different approaches to measure exposure and outcome, allowing us to triangulate evidence across study samples with potentially different sources of misclassification. For example, in the *Clinical Sample* where BMI was measured by professionals, we found similar associations between frequent nighttime smartphone use and BMI as in the two study samples, in which BMI was self-reported. In the *Population Sample*, we found similar associations between BMI and both self-reported and latent clusters of nighttime smartphone use, respectively. These findings may indicate that differential misclassification is of little concern.

In the *Clinical Sample*, cardiometabolic biomarkers were measured from non-fasting blood samples, which is recommended to use as the clinical standard in Denmark^63^. Previously, non-fasting lipid profiles have been perceived as less accurate measurements than fasting lipid profiles when assessing cardiovascular risk, thus leading to potential misclassification of cardiovascular risk^64,65^. However, several population-based prospective studies have provided evidence to use non-fasting lipid profiles, as they have shown similar cardiovascular risk assessments compared to fasting lipid profiles^63^. Thus, using non-fasting lipid profiles may not explain the lack of associations between nighttime smartphone use and cardiometabolic risk markers.

The study samples reflect selected populations and may not represent the general population. Even though the *Population Sample* and the *Clinical Sample* were randomly drawn from the general population to ensure representativeness, they are challenged by a relatively low level of participation. The same issue with representativeness is also a concern in the *Citizen Science Sample,* as it is a self-selected sample^22^. We have previously performed sociodemographic comparisons between each study sample in the *SmartSleep Study* and the general adult population to address the concern^47^. We found that more females, middle-aged individuals, and individuals with a higher educational level were more likely to participate in the *SmartSleep Study.* In the *Citizen Science Sample,* only 15% of the participants participated in the follow-up study. Thus, loss of follow-up may also impact our results. We found that more women and slightly older participants were more likely to participate in the follow-up study. As women tend to have more frequent nighttime smartphone use and older participants tend to have less frequent nighttime smartphone use, this may have impacted our results in different directions. Nevertheless, the lack of association between nighttime smartphone use and changes in BMI suggests that loss of follow-up is of lesser concern. Due to the study design, a few night workers were included in the study populations (N < 1%). We find it unlikely that the inclusion of this group impacted our results.

We assigned individuals to a specific latent cluster based on their maximum posterior probabilities to ease the interpretability of the findings. However, we thereby reduced the complexity of the latent cluster assignment composition. Instead, future studies may benefit from using compositional data analysis methods to include the posterior probabilities of individuals partially belonging to several latent classes as compositional covariates in the models^66^.

## Conclusion

We found that frequent nighttime smartphone use is associated with overweight, obesity, and higher BMI across diverse population samples. Assuming a causal basis for these associations, nighttime smartphone use may negatively impact metabolism and weight. Thus, nighttime smartphone use may be a potential target point for public health interventions to reduce overweight and obesity at population levels. Nevertheless, larger longitudinal studies using cardiometabolic risk markers are warranted to elucidate the complex relationship between nighttime smartphone use, sleep, overweight, and cardiometabolic dysfunction.

### Supplementary Information

Supplementary Information.

## Data Availability

The data underlying this article cannot be shared publicly due to the privacy of individuals that participated in the study. The data will be shared on reasonable request to the principal investigator of the SmartSleep project Professor Naja Hulvej Rod (nahuro@sund.ku.dk).
