# Supplementary material for: Self-reported and tracked nighttime smartphone use and their association with overweight and cardiometabolic risk markers
Source: Sci Rep. 2024 Feb 28;14:4861. doi: 10.1038/s41598-024-55349-2 (PMC10902390; doi:10.1038/s41598-024-55349-2)
Supplement: Supplementary file 1 — Supplementary Information. [file 41598_2024_55349_MOESM1_ESM.docx]

**Self-reported and Tracked Nighttime Smartphone Use and their Association with Overweight and Cardiometabolic Risk Markers**

Thea Otte Andersen^1*^, Christoffer Sejling^2^, Andreas Kryger Jensen^2^, Agnete Skovlund Dissing^3^, Elin Rosenbek Severinsen^1^, Henning Johannes Drews^1^, Thorkild IA Sørensen^1^, Tibor V Varga^1^, Naja Hulvej Rod^1^

**Supplementary Text S1:** Description of the four latent clusters of individuals’ nighttime smartphone use

The four latent clusters of individuals’ nighttime smartphone use are characterized accordingly:

1) The *non-user* is characterized by having most *non-use* nights (70%) and some nights with sleep onset use (19%), sleep offset use (8%), and continuous use (4%).

*2)* The *sleep onset user* is characterized by most nights of *sleep onset use* (44%) and non-use (35%) and some nights with *sleep offset use* (14%) and *continuous use* (7%)

3) The *sleep offset user* is characterized by most nights of *sleep offset use* (60%) and some nights with *continuous use* (15%)*, non-use* (14%), and *sleep onset use* (11%).

4) The *all-user* is characterized by having a mix of nights with *continuous use (*32%), *sleep onset use* (39%) and *sleep offset use* (23%), and *non-use* (5%).

| **Supplementary Table S1:** Overview of Cardiometabolic risk markers | | |
| --- | --- | --- |
| **Clinical data** | **Definition** | **Cut-off points for metabolic dysfunction in young females** |
| Height | Measured standing without shoes and heals touching the wall in cm |  |
| Weight | Measured in light clothes without shoes in kilograms |  |
| BMI | Measured based on clinical height and weight and calculated as weight in kilograms divided by height in meters squared. | Overweight: BMI between 25 and 29.9 kg/m^2^  Obesity: BMI ≥30 kg/m^2^ |
| Waist-hip ratio | The ratio of the waist circumference to the hip circumference.  Waist circumference was measured at the end of a normal expiration in a standing relaxed position.  Hip circumference was measured at the widest horizontal level around the hip. | ≥0.85 |
| Blood pressure | Measured seated with non-cross legs in both arms. Measurements on both arms were performed and the reported blood pressure is the average of the consecutive second and third measurements on the arm with the highest systolic blood pressure. Measurements were performed at approx. two minutes intervals. Measurements were performed using an automated blood pressure cuff at the appropriate size. | Systolic > 140 mmHg  Diastolic > 90 mmHg |
| **Metabolic biomarkers** | | |
| Triglycerides | Measured from non-fasting venous blood using Li-heparin tubes | ≥2 mmol/L |
| High-density lipoprotein cholesterol (HDL-C) | Measured from non-fasting venous blood using Li-heparin tubes | <1.2 mmol/L |
| Low-density lipoprotein cholesterol (LDL-C) | Measured from non-fasting venous blood using Li-heparin tubes | ≥4.3 mmol/L |
| Very low-density lipoprotein cholesterol (VLDL-C) | Measured from non-fasting venous blood using Li-heparin tubes | ≥1.7 mmol/L |
| Total cholesterol | The overall amount of cholesterol in the blood (HDL-C, LDL-C, VLDL-C) | >5 mmol/L |
| Glycated hemoglobin (HbA1c) | Measured from non-fasting venous blood using K2-EDTA tubes | > 48 mmol/mol |

**Supplementary Table S2:** Weights calculated based on the distribution in the Danish Population.

We show the distributions of age, sex, and geographical region in the Danish population and in the *Baseline* *Citizen Science Sample (*Supplemental Table 2A)*,* *Citizen Science* *Follow-up Sample (*Supplemental Table 2B), and *Population Sample* (Supplemental Table 2C). These distributions were used to calculate weights used in the analyses to make the samples more representative. E.g., in the *Baseline Citizen Science Sample*, 62% of the population were females, and in the Danish population, only 50% were women. This means that women had a smaller weight, while men had a higher weight in the *Baseline Citizen Science Sample*.

| **Supplementary Table S2A**. Distributions of age, sex, and geographical region in the Danish population (aged 16+) and in the *Baseline Citizen Science Sample* | | |
| --- | --- | --- |
|  | Danish population aged 16+,  1 November 2018  N= 4,780,013 | *Baseline Citizen Science Sample*  N=25,135 |
|  | **%** | **%** |
| **Age group** |  |  |
| 16-19 | 6 | 6 |
| 20-24 | 8 | 8 |
| 25-29 | 8 | 11 |
| 30-34 | 7 | 9 |
| 35-39 | 7 | 9 |
| 40-44 | 8 | 11 |
| 45-49 | 8 | 11 |
| 50-44 | 9 | 12 |
| 55-59 | 8 | 9 |
| 60-64 | 7 | 7 |
| 65+69 | 7 | 5 |
| 70-74 | 7 | 3 |
| 75-79 | 5 | 1 |
| 80+ | 5 | 0 |
| **Sex** |  |  |
| Male | 50 | 38 |
| Female | 50 | 62 |
| **Geographical region** |  |  |
| Capital Region | 32 | 43 |
| Region Zealand | 14 | 12 |
| Region of Southern Denmark | 23 | 17 |
| Central Denmark Region | 23 | 21 |
| North Denmark Region | 10 | 7 |

| **Supplementary Table S2B**. Distributions of age, sex, and geographical region in the Danish population (aged 16+) and in the *Citizen Science Follow-up Sample* | | |
| --- | --- | --- |
|  | Danish population aged 16+,  1 January 2020  N=4,801,877 | *Citizen Science Follow-up Sample*  N=1,885 |
|  | % | % |
| **Age group** |  |  |
| 16-20 | 8 | 2 |
| 21-25 | 9 | 8 |
| 26-30 | 9 | 11 |
| 31-35 | 8 | 8 |
| 36-40 | 7 | 9 |
| 41-45 | 8 | 11 |
| 46-50 | 8 | 13 |
| 51-55 | 9 | 13 |
| 56-60 | 8 | 10 |
| 61-65 | 7 | 7 |
| +65 | 19 | 6 |
| **Sex** |  |  |
| Male | 50 | 35 |
| Female | 50 | 65 |
| **Region** |  |  |
| Capital Region | 32 | 50 |
| Region Zealand | 14 | 13 |
| Region of Southern Denmark | 21 | 14 |
| Central Denmark Region | 23 | 18 |
| North Denmark Region | 10 | 6 |

| **Supplementary Table S2C:** Distributions of age, sex, and geographical region in the Danish population (aged 18-50) and in the *Population Sample* | | |
| --- | --- | --- |
|  | Danish population aged 18-50,  1 January 2020  N=2,441,447 | *Population Sample*  N=4,522 |
|  | % | % |
| **Age group** |  |  |
| 18-22 | 15 | 10 |
| 23-27 | 17 | 14 |
| 28-32 | 15 | 13 |
| 33-37 | 14 | 13 |
| 38-42 | 14 | 14 |
| 43-47 | 16 | 20 |
| 48+ | 9 | 16 |
| **Sex** |  |  |
| Male | 51 | 35 |
| Female | 49 | 65 |
| **Region** |  |  |
| Capital Region | 35 | 34 |
| Region Zealand | 13 | 11 |
| Region of Southern Denmark | 20 | 19 |
| Central Denmark Region | 23 | 26 |
| North Denmark Region | 10 | 9 |

**Supplementary Text S2:** Box-Cox-Cole and Green (BCCG) model

More details about the theoretical structure of the GAMLSS regression model framework are provided in Rigby et al. 2005.^45^ The BCCG model is also included in the class of Box-Cox Symmetric distribution,^58^ which is a family of distributions well-suited for modeling skewed data on the original scale, allowing for straightforward parameter interpretations. In the BCCG model, the location parameter parameterized by the center of the distribution is often equal to or close to the median. We employ a BCCG model structure such that the location and shape parameters are modeled linearly, while the scale parameter is modeled log-linearly. In describing the analysis results, fitted centers and center contrasts of interest are translated into means and mean contrasts. Assuming that the scale and shape parameters are constant, it holds for the Box-Cox Symmetric distributions that the mean scales linearly in the center of the distribution.^58^ In particular, the mean is a monotone function of the median mapping zero to zero. Hence, the mean contrasts are uniquely defined from the center contrasts in this situation. As a result, confidence regions for the mean can be calculated directly from the model parameter confidence regions by use of the center-to-mean mapping, and *P* values for the significance tests of mean contrasts can be taken directly from the significance tests of model parameters. The constant scale and shape parameter assumption can be ensured by including only an intercept in their respective linear predictors.

| **Supplementary Table S3.** Characteristics in the *Population Sample* stratified on self-reported nighttime smartphone use among 4,522 adults in Denmark | | | | | |
| --- | --- | --- | --- | --- | --- |
|  |  | **Frequency of self-reported nighttime smartphone use** | | | |
|  | **Total**  N=4,522 | **Never**  N=1,268 (28%) | **A few nights a month**  N=2,029 (45%) | **A few nights a week**  N=905 (20%) | **Every night or almost every night**  N=320 (7%) |
| **Age,** mean (SD) | 36.6 (9.7) | 39.2 (9.2) | 36.2 (9.7) | 34.4 (9.7) | 34.5 (9.7) |
| **Sex,** N (%) | | | | | |
| Female | 2,939 (65) | 720 (57) | 1,335 (66) | 644 (71) | 239 (75) |
| Male | 1,583 (35) | 548 (43) | 694 (34) | 261 (28) | 81 (25) |
| **Educational level,** N (%) | | | | | |
| Primary school | 272 (6) | 57 (5) | 114 (6) | 77 (9) | 24 (7) |
| Upper secondary school | 599 (13) | 119 (9) | 279 (14) | 154 (17) | 47 (14) |
| Technical/Vocational education | 800 (18) | 247 (20) | 332 (17) | 153 (17) | 68 (21) |
| Short-cycle higher education | 388 (9) | 127 (10) | 165 (8) | 72 (8) | 24 (8) |
| Medium-cycle higher education | 1,297 (29) | 371 (29) | 597 (29) | 244 (27) | 85 (27) |
| Long-cycle higher education | 1,052 (23) | 310 (24) | 495 (24) | 186 (21) | 61 (19) |
| Other | 114 (2) | 36 (3) | 47 (2) | 18 (2) | 13 (4) |
| **Occupational level,** N (%) | | | | | |
| Student | 732 (16) | 128 (10) | 341 (17) | 199 (22) | 64 (28) |
| Employed | 3,282 (73) | 1,042 (82) | 1,483 (73) | 580 (64) | 177(51) |
| Unemployed | 155 (3) | 30 (2) | 71 (4) | 43 (5) | 11 (4) |
| Long-term sick leave | 84 (2) | 17 (1) | 29 (1) | 20 (2) | 18 (2) |
| Outside labor market | 73 (2) | 18 (1) | 23 (1) | 17 (2) | 15 (8) |
| Other | 196 (4) | 34 (3) | 83 (4) | 44 (5) | 35 (6) |

| **Supplementary Table S4.** Characteristics in the *Clinical Sample* among 242 women^1^ | |
| --- | --- |
|  | **Total**  N=242 |
| **Age,** mean (SD) | 23.2 (2.1) |
| **Educational level**^2^**,** N (%) |  |
| Primary school or other | 25 (10) |
| Upper secondary school | 115 (48) |
| Technical/Vocational education/Short-cycle higher education | 14 (6) |
| Medium-cycle higher education | 66 (27) |
| Long-cycle higher education | 22 (9) |
| **Occupational status,** N (%) |  |
| Student | 156 (64) |
| Employed | 68 (28) |
| Other^3^ | 18 (7) |
| **Nighttime smartphone use,** N (%) |  |
| Never | 27 (11) |
| A few nights a month | 120 (50) |
| A few nights a week | 66 (27) |
| Every night or almost every night | 29 (12) |
| ^1^Due to small numbers and data protection, we do not show the distributions stratified on self-reported nighttime smartphone use.  ^2^ Categories in educational level are merged due to small numbers.  ^3^ ‘Other’ category includes unemployed, outside labor market, long-term sick leave and other | |

| **Supplementary Table S5.** Association between self-reported sleep quality and obesity^1^ in the *Baseline Citizen Science Sample*, the *Population Sample* and pooled | | | |
| --- | --- | --- | --- |
|  | **Citizen Science Sample**  N=25,074 | **Population Sample**  N=4,522^2^ | **Pooled** |
|  | OR (95% CI) | OR (95% CI) | OR (95% CI) |
| **Sleep quality** | |  |  |
| Q1 | Ref. | Ref. | Ref. |
| Q2 | 1.07 (0.97;1.18) | 1.06 (0.81;1.38) | 1.07 (0.97;1.17) |
| Q3 | 1.15 (1.03;1.27) | 1.02 (0.79;1.32) | 1.13 (1.03;1.25) |
| Q4 | 1.33 (1.20;147) | 1.34 (0.99;1.81) | 1.33 (1.21;1.47) |
| ^1^ Outcome variable: obesity defined as Body Mass Index≥30kg/m^2^  Logistic regression models adjusted for age, gender/sex, educational level, occupational status, and nighttime smartphone use were applied.  Models were weighted by sample weights for *Population Sample* and *Baseline Citizen Science Sample*  OR: odds ratio, 95% CI: 95% confidence intervals, ref: reference group | | | |

| **Supplementary Table S6.** Association between self-reported and tracked nighttime smartphone use and obesity^1^ in the *Baseline Citizen Science Sample*, the *Population Sample* and pooled | | | |
| --- | --- | --- | --- |
|  | **Citizen Science Sample**  N=25,074 | **Population Study**  N=4,522^2^ | **Pooled** |
|  | OR (95% CI) | OR (95% CI) | OR (95% CI) |
| **Self-reported nighttime smartphone use** | |  |  |
| Never | Ref. | Ref. | Ref. |
| A few nights a month | 1.17 (1.08-1.27) | 1.13 (0.89-1.43) | 1.17 (1.08;1.26) |
| A few nights a week | 1.36 (1.21-1.53) | 1.45 (1.12-1.88) | 1.37 (1.24;1.53) |
| Every night or almost every night | 1.65 (1.40-1.95) | 1.56 (1.09-2.22) | 1.63 (1.41;1.90) |
| **Latent clusters of tracked nighttime smartphone use** | |  |  |
| Non-user | *-* | Ref. | *-* |
| Sleep onset user | *-* | 1.41 (1.07-1.85) | *-* |
| Sleep offset user | *-* | 1.66 (1.28-2.14) | *-* |
| All-user | *-* | 1.37 (0.99-1.89) | *-* |
| ^1^ Outcome variable: obesity defined as Body Mass Index≥30kg/m^2^  ^2^ Latent clusters of tracked nighttime smartphone use: Population Sample: N=3,222  Logistic regression models adjusted for age, gender/sex, educational level, and occupational status were applied.  Models were weighted by sample weights for *Population Sample* and *Baseline Citizen Science Sample*  OR: odds ratio, 95% CI: 95% confidence intervals, ref: reference group | | | |

| **Supplementary Table S7.** Sensitivity analysis on the association between self-reported and tracked nighttime smartphone use and overweight^1^ in the *Population Sample* with further adjusting for physical activity | |
| --- | --- |
|  | **Population Sample**  N=4,522^2^ |
|  | OR (95% CI) |
| **Self-reported nighttime smartphone use** | |
| Never | Ref. |
| A few nights a month | 1.21 (1.03-1.43) |
| A few nights a week | 1.48 (1.21-1.82) |
| Every night or almost every night | 1.54 (1.17-2.05) |
| **Latent clusters of tracked nighttime smartphone use** | |
| Non-user | Ref. |
| Sleep onset user | 1.02 (0.83;1.25) |
| Sleep offset user | 1.14 (0.93;1.39) |
| All-user | 1.34 (1.05;1.71) |
| ^1^ Outcome variable: overweight defined as Body Mass index≥25kg/m^2^  ^2^ Latent clusters of nighttime smartphone use: Population Sample: N=3,222  Logistic regression models adjusted for age, sex, educational level, occupational status, and physical activity were applied.  Models were weighted by sample weights for *Population Sample*  OR: odds ratio, 95% CI: 95% confidence intervals, ref: reference group | |
